# Crystal structure of di­chlorido­bis­(methyl isonicotinate-κ*N*)copper(II)

**DOI:** 10.1107/S205698901500729X

**Published:** 2015-04-18

**Authors:** Elaheh Ahadi, Hassan Hosseini-Monfared, Peter Mayer

**Affiliations:** a Department of Chemistry, University of Zanjan 45195-313, Zanjan, Islamic Republic of Iran; b Ludwig-Maximilians-Universität, Department Chemie, Butenandtstrasse 5–13, 81377 München, Germany

**Keywords:** crystal structure, square-planar copper(II) complex, methyl isonicotinate

## Abstract

In the title compound, [CuCl_2_(C_7_H_7_NO_2_)_2_], the square-planar-coordinated Cu^II^ ion lies on a centre of symmetry and is bonded to two monodentate methyl­isonicotinate ligands through their N atoms and by two chloride ligands. The mol­ecules pack in a herringbone pattern. Perpendicular to [100] there are weak inter­molecular C—H⋯Cl and C—H⋯O contacts. Along [100] there are infinite chains of edge-sharing octa­hedra linked through the chlorido ligands

## Related literature   

For related structures, see: Vitorica-Yrezabal *et al.* (2011 ▸); Laing & Carr (1971 ▸); Chen & Mak (2006 ▸); Ge *et al.* (2006 ▸); Chen *et al.* (2011 ▸); Ma *et al.* (2010 ▸). For background to isonicotinate, see: Zhou *et al.* (2006 ▸); Bera *et al.* (2001 ▸); Cotton *et al.* (2007 ▸); Tella *et al.* (2014 ▸). For the synthesis of 4-(5-phenyl-1,3,4-oxa­diazol-2-yl)pyridine, used in the preparation, see: Kangani & Day (2009 ▸).
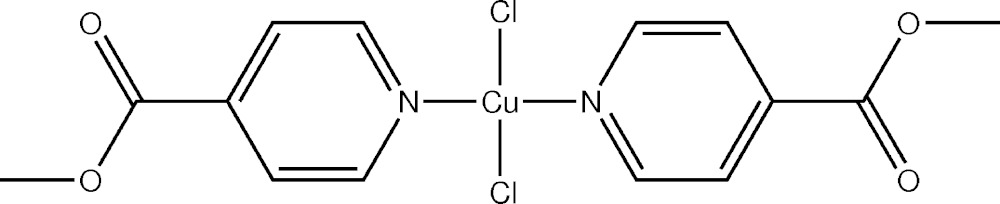


## Experimental   

### Crystal data  

[CuCl_2_(C_7_H_7_NO_2_)_2_]
*M* *_r_* = 408.71
Monoclinic, 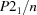

*a* = 3.7792 (4) Å
*b* = 29.891 (4) Å
*c* = 7.0139 (8) Å
β = 94.036 (10)°
*V* = 790.36 (16) Å^3^
*Z* = 2
Mo *K*α radiation
μ = 1.74 mm^−1^
*T* = 173 K
0.50 × 0.05 × 0.04 mm

### Data collection  

Oxford Diffraction Xcalibur 3 diffractometer
Absorption correction: multi-scan (*CrysAlis PRO*; Oxford Diffraction, 2011 ▸) *T*_min_ = 0.775, *T*_max_ = 1.000
4265 measured reflections
1617 independent reflections
1404 reflections with *I* > 2σ(*I*)
*R*_int_ = 0.034

### Refinement  

*R*[*F* ^2^ > 2σ(*F* ^2^)] = 0.036
*wR*(*F* ^2^) = 0.091
*S* = 1.09
1617 reflections
107 parameters
H-atom parameters constrained
Δρ_max_ = 0.53 e Å^−3^
Δρ_min_ = −0.69 e Å^−3^

### 

Data collection: *CrysAlis PRO* (Oxford Diffraction, 2011 ▸); cell refinement: *CrysAlis PRO*; data reduction: *CrysAlis PRO*; program(s) used to solve structure: *SIR97* (Altomare *et al.*, 1999 ▸); program(s) used to refine structure: *SHELXL2014* (Sheldrick, 2008 ▸, 2015 ▸); molecular graphics: *ORTEPIII* (Burnett & Johnson, 1996 ▸); software used to prepare material for publication: *PLATON* (Spek, 2009 ▸).

## Supplementary Material

Crystal structure: contains datablock(s) I, global. DOI: 10.1107/S205698901500729X/cq2014sup1.cif

**Fig. 1.**
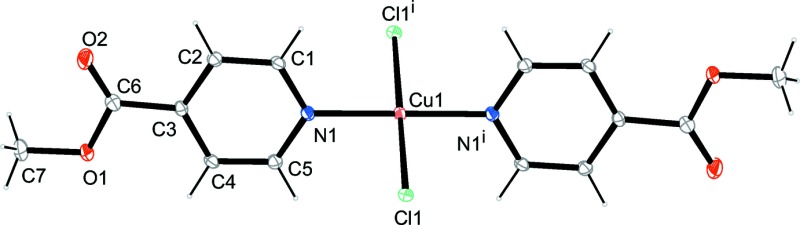
The molecular structure of the title compound (ellipsoids drawn at the 30% probability level). Symmetry code: i = 1 - x, -y, 1 - z. Non-labelled non-hydrogen atoms have been generated by symmetry i.

Structure factors: contains datablock(s) I. DOI: 10.1107/S205698901500729X/cq2014Isup2.hkl

x y z . DOI: 10.1107/S205698901500729X/cq2014fig1.tif
The mol­ecular structure of the title compound (ellipsoids drawn at the 30% probability level). Symmetry code: i = 1 − *x*, −*y*, 1 − *z*. Non-labelled non-hydrogen atoms have been generated by symmetry i.

**Fig. 2.**
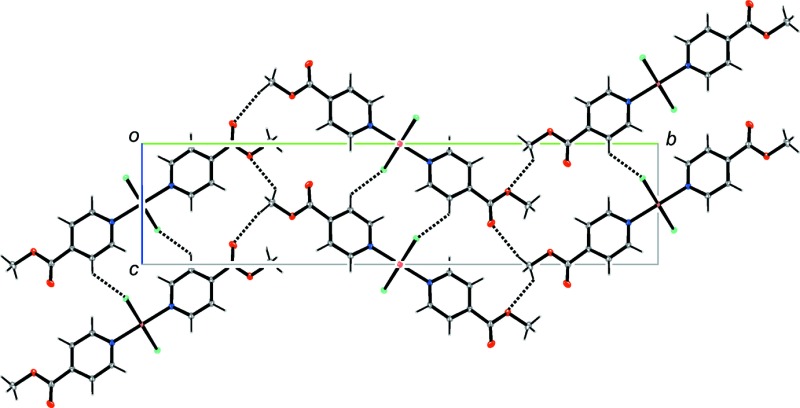
The unit cell viewed along [100] (ellipsoids drawn at the 50% probability level). Intermolecular C—H···Cl and C—H···O contacts are indicated by dashed lines.

. DOI: 10.1107/S205698901500729X/cq2014fig2.tif
The unit cell viewed along [100] (ellipsoids drawn at the 50% probability level). Inter­molecular C—H⋯Cl and C—H⋯O contacts are indicated by dashed lines.

**Fig. 3.**
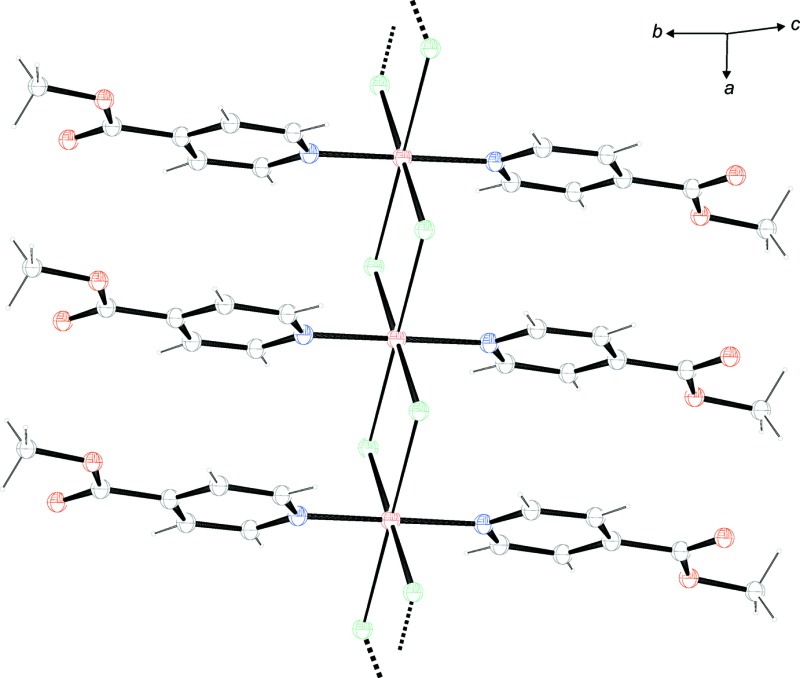
Infinite strands along [100] formed by intermolecular Cu—Cl bonds (thin bond diameter) (drawn at the 30% ellipsoid probability level).

. DOI: 10.1107/S205698901500729X/cq2014fig3.tif
Infinite strands along [100] formed by inter­molecular Cu—Cl bonds (thin bond diameter) (drawn at the 30% ellipsoid probability level).

CCDC reference: 1059032

Additional supporting information:  crystallographic information; 3D view; checkCIF report

## Figures and Tables

**Table 1:** Hydrogen-bond geometry (, )

*D*H*A*	*D*H	H*A*	*D* *A*	*D*H*A*
C2H2Cl1^i^	0.95	2.83	3.517(3)	130
C7H7*B*O2^ii^	0.98	2.53	3.470(4)	161
C7H7*C*O1^iii^	0.98	2.58	3.298(4)	130

Symmetry codes: (i) 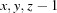; (ii) 
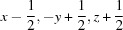
; (iii) 
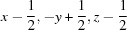
.

## supplementary crystallographic information

### S1. Comment

Isonicotinate is a versatile ditopic ligand that has been used for various applications. The copper(II) isonicotinate (Cu(4–C_5_H_4_N-COO)_2_(H_2_O)_4_) coordination polymer has been explored as a sorbent in flow injection solid-phase extraction for determination of trace polycyclic aromatic hydrocarbons in environmental matrices [Zhou *et al.* (2006)]. The incorporation of both dinuclear (*M*_2_) and mononuclear (*M*') units into molecular squares has been achieved by reacting a triply bonded Re_2_(II,II) complex possessing two *cis* isonicotinate donor ligands with Pt(II) containing molecules with substitutionally labile *cis* trifluoromethanesulfonate groups [Bera *et al.* (2001)]. Quadruply bonded Mo_2_^4+^ species having isonicotinate ligands bound through the carboxylate group have been designed to act as 'anglers' by luring metal-containing Lewis acids to bind to the N-pyridyl group [Cotton *et al.* (2007)]. The copper-isonicotinate metal-organic frameworks [Cu(INA)_2_] (INA = isonicotinate) (MOFs) have been prepared simply by mixing and heating solid reactants without milling. The adsorption of fluorescein dye on the as-synthesized [Cu(INA)_2_] has also been investigated [Tella *et al.* (2014)].

The molecular structure of the title compound possessing a crystallographic centre of inversion is depicted in Fig. 1. The first coordination sphere of the Cu1 centre consists of two nitrogen atoms, from the isoniconate ligands, at a distance of 2.025 (2) Å and two chlorido ligands at a distance of 2.2962 (7) Å. The various N1—Cu1—Cl1 angles are close to 90° resulting in a square-planar first coordination sphere of Cu1. By coordination of two additional chlorido ligands from adjacent complexes at a distance of 2.9215 (7) Å from the Cu1 centre, the coordination sphere is expanded to a distorted octahedron. The pyridine ring and the Cu1—Cl1 bond are not coplanar, but enclose an angle of 59.00 (9)°. The Cu—N1 bond and the plane of the pyridine ring deviate from coplanarity by an angle of 5.05 (11)°.

The herringbone pattern of the packing of the title compound is shown in Fig. 2. Perpendicular to [100] there are weak intermolecular C—H···Cl and C—H···O contacts with donor-acceptor distances of 3.517 (3) Å, 3.470 (4) Å and 3.298 (4) Å (C2···Cl1, C7···O2 and C7···O1, respectively). Along [100] there are infinite chains of edge-sharing octahedra linked through the chlorido ligands (Fig.3). This structural feature is found in many copper complexes consisting of two chlorido ligands and two pyridine derivatives, *e.g.* Vitorica-Yrezabel *et al.* (2011), Laing *et al.* (1971), Chen *et al.* (2006), Ge *et al.* (2006), Chen *et al.* (2011), Ma *et al.* (2010). Each bridging chlorido ligand has a short (2.2962 (7) Å) and a longer (2.9215 (7) Å) bond distance to the two adjacent Cu-centers. The bond angle at the bridging chloride ions is 92.03 (2)°. A consequence of the formation of the strands along [100] is π-stacking between adjacent pyridine rings. The average distance between the centres of gravity of adjacent rings is *a* = 3.7792 Å.

### S2. Experimental

The compound 4-(5-phenyl-1,3,4-oxadiazol-2-yl)pyridine (0.04 g, 0.18 mmol, synthesised by a previously reported method [Kangani *et al.* (2009)], and copper(II) chloride dihydrate (0.015 g, 0.09 mmol) were placed in the main arm of a branched tube. Methanol (13 ml) was carefully added to fill the arms, the tube was sealed and the reagent-containing arm immersed in an oil bath at 60 °C while the other arm was kept at ambient temperature. After two weeks, green, needle shaped crystals were deposited in the cooler arm. The crystals were filtered, washed with methanol and air dried. Yield 19% (7.0 mg).

### S3. Refinement

All hydrogen atoms were positioned geometrically and treated as riding on their parent atoms (aromatic C—H = 0.95 Å, methyl C—H = 0.98 Å, *U*_iso_(H) = 1.2*U*_eq_(C, aromatic), *U*_iso_(H) = 1.5*U*_eq_(C, methyl)). The methyl group was allowed to rotate along the C–O bond to best fit the experimental electron density.

### Crystal data

**Table:**

[CuCl_2_(C_7_H_7_NO_2_)_2_]	*F*(000) = 414
*M**_r_* = 408.71	*D*_x_ = 1.717 Mg m^−^^3^
Monoclinic, *P*2_1_/*n*	Mo *K*α radiation, λ = 0.71073 Å
*a* = 3.7792 (4) Å	Cell parameters from 1293 reflections
*b* = 29.891 (4) Å	θ = 4.5–26.3°
*c* = 7.0139 (8) Å	µ = 1.74 mm^−^^1^
β = 94.036 (10)°	*T* = 173 K
*V* = 790.36 (16) Å^3^	Rod, green
*Z* = 2	0.50 × 0.05 × 0.04 mm

### Data collection

**Table:**

Oxford Diffraction Xcalibur 3 diffractometer	1617 independent reflections
Radiation source: fine-focus sealed tube	1404 reflections with *I* > 2σ(*I*)
Detector resolution: 15.9809 pixels mm^-1^	*R*_int_ = 0.034
ω scans	θ_max_ = 26.4°, θ_min_ = 4.5°
Absorption correction: multi-scan (*CrysAlis PRO*; Oxford Diffraction, 2011)	*h* = −4→4
*T*_min_ = 0.775, *T*_max_ = 1.000	*k* = −33→37
4265 measured reflections	*l* = −8→6

### Refinement

**Table:**

Refinement on *F*^2^	0 restraints
Least-squares matrix: full	Hydrogen site location: inferred from neighbouring sites
*R*[*F*^2^ > 2σ(*F*^2^)] = 0.036	H-atom parameters constrained
*wR*(*F*^2^) = 0.091	*w* = 1/[σ^2^(*F*_o_^2^) + (0.0388*P*)^2^ + 0.6044*P*] where *P* = (*F*_o_^2^ + 2*F*_c_^2^)/3
*S* = 1.09	(Δ/σ)_max_ < 0.001
1617 reflections	Δρ_max_ = 0.53 e Å^−^^3^
107 parameters	Δρ_min_ = −0.69 e Å^−^^3^

### Special details

**Table:**

Experimental. Absorption correction: CrysAlisPro (Oxford Diffraction, 2011) Empirical absorption correction using spherical harmonics, implemented in SCALE3 ABSPACK scaling algorithm.
Geometry. All e.s.d.'s (except the e.s.d. in the dihedral angle between two l.s. planes) are estimated using the full covariance matrix. The cell e.s.d.'s are taken into account individually in the estimation of e.s.d.'s in distances, angles and torsion angles; correlations between e.s.d.'s in cell parameters are only used when they are defined by crystal symmetry. An approximate (isotropic) treatment of cell e.s.d.'s is used for estimating e.s.d.'s involving l.s. planes.

### Fractional atomic coordinates and isotropic or equivalent isotropic displacement parameters (Å^2^)

**Table:**

	*x*	*y*	*z*	*U*_iso_*/*U*_eq_	
Cu1	0.5000	0.0000	0.5000	0.01825 (17)	
Cl1	0.91406 (17)	0.03090 (2)	0.71647 (9)	0.01768 (18)	
O2	0.3701 (7)	0.17679 (8)	−0.1593 (3)	0.0414 (7)	
O1	0.1672 (6)	0.20909 (6)	0.0994 (3)	0.0291 (5)	
N1	0.4707 (6)	0.05771 (7)	0.3486 (3)	0.0168 (5)	
C1	0.5365 (7)	0.05831 (9)	0.1633 (4)	0.0180 (6)	
H1	0.6172	0.0316	0.1070	0.022*	
C2	0.4918 (7)	0.09617 (9)	0.0505 (4)	0.0197 (6)	
H2	0.5444	0.0955	−0.0800	0.024*	
C3	0.3693 (7)	0.13491 (9)	0.1308 (4)	0.0174 (6)	
C4	0.3076 (8)	0.13494 (9)	0.3240 (4)	0.0188 (6)	
H4	0.2278	0.1613	0.3837	0.023*	
C5	0.3647 (8)	0.09584 (9)	0.4276 (4)	0.0202 (6)	
H5	0.3272	0.0961	0.5601	0.024*	
C6	0.3063 (8)	0.17503 (10)	0.0051 (4)	0.0236 (7)	
C7	0.0886 (11)	0.24908 (11)	−0.0132 (5)	0.0384 (9)	
H7A	0.3090	0.2613	−0.0577	0.058*	
H7B	−0.0224	0.2714	0.0657	0.058*	
H7C	−0.0739	0.2416	−0.1236	0.058*	

### Atomic displacement parameters (Å^2^)

**Table:**

	*U*^11^	*U*^22^	*U*^33^	*U*^12^	*U*^13^	*U*^23^
Cu1	0.0235 (3)	0.0123 (3)	0.0184 (3)	−0.00169 (19)	−0.0021 (2)	0.00175 (18)
Cl1	0.0186 (3)	0.0170 (4)	0.0175 (3)	−0.0002 (3)	0.0016 (3)	−0.0023 (2)
O2	0.0712 (19)	0.0277 (13)	0.0274 (13)	0.0115 (12)	0.0191 (13)	0.0092 (10)
O1	0.0465 (14)	0.0169 (11)	0.0239 (12)	0.0100 (10)	0.0035 (10)	0.0022 (9)
N1	0.0175 (12)	0.0144 (12)	0.0185 (12)	0.0009 (9)	0.0002 (9)	−0.0003 (9)
C1	0.0201 (14)	0.0150 (14)	0.0192 (14)	0.0007 (11)	0.0038 (11)	−0.0031 (11)
C2	0.0220 (15)	0.0190 (15)	0.0185 (14)	−0.0010 (11)	0.0048 (11)	−0.0006 (11)
C3	0.0169 (14)	0.0141 (14)	0.0210 (14)	−0.0016 (10)	0.0003 (11)	0.0010 (11)
C4	0.0212 (14)	0.0146 (14)	0.0211 (15)	0.0005 (11)	0.0043 (11)	−0.0029 (11)
C5	0.0242 (15)	0.0186 (14)	0.0184 (14)	−0.0006 (12)	0.0049 (12)	−0.0007 (11)
C6	0.0254 (16)	0.0192 (16)	0.0264 (17)	0.0002 (12)	0.0023 (13)	0.0000 (12)
C7	0.056 (2)	0.0217 (17)	0.037 (2)	0.0133 (16)	0.0034 (17)	0.0075 (14)

### Geometric parameters (Å, º)

**Table:**

Cu1—N1	2.025 (2)	C1—H1	0.9500
Cu1—N1^i^	2.025 (2)	C2—C3	1.382 (4)
Cu1—Cl1	2.2962 (7)	C2—H2	0.9500
Cu1—Cl1^i^	2.2962 (7)	C3—C4	1.391 (4)
Cu1—Cl1^ii^	2.9215 (7)	C3—C6	1.498 (4)
Cu1—Cl1^iii^	2.9215 (7)	C4—C5	1.385 (4)
O2—C6	1.196 (4)	C4—H4	0.9500
O1—C6	1.341 (3)	C5—H5	0.9500
O1—C7	1.452 (4)	C7—H7A	0.9800
N1—C1	1.340 (4)	C7—H7B	0.9800
N1—C5	1.341 (3)	C7—H7C	0.9800
C1—C2	1.385 (4)		
			
N1—Cu1—N1^i^	180.00 (6)	C3—C2—C1	118.9 (3)
N1—Cu1—Cl1	90.79 (7)	C3—C2—H2	120.6
N1^i^—Cu1—Cl1	89.21 (7)	C1—C2—H2	120.6
N1—Cu1—Cl1^i^	89.21 (7)	C2—C3—C4	118.8 (3)
N1^i^—Cu1—Cl1^i^	90.79 (7)	C2—C3—C6	118.4 (3)
Cl1—Cu1—Cl1^i^	180.0	C4—C3—C6	122.8 (3)
N1—Cu1—Cl1^ii^	90.70 (7)	C5—C4—C3	118.7 (3)
N1^i^—Cu1—Cl1^ii^	89.29 (7)	C5—C4—H4	120.7
Cl1—Cu1—Cl1^ii^	87.97 (2)	C3—C4—H4	120.7
Cl1^i^—Cu1—Cl1^ii^	92.03 (2)	N1—C5—C4	122.7 (3)
N1—Cu1—Cl1^iii^	89.30 (7)	N1—C5—H5	118.6
N1^i^—Cu1—Cl1^iii^	90.71 (7)	C4—C5—H5	118.6
Cl1—Cu1—Cl1^iii^	92.03 (2)	O2—C6—O1	123.7 (3)
Cl1^i^—Cu1—Cl1^iii^	87.97 (2)	O2—C6—C3	124.6 (3)
Cl1^ii^—Cu1—Cl1^iii^	180.0	O1—C6—C3	111.7 (2)
C6—O1—C7	115.4 (2)	O1—C7—H7A	109.5
C1—N1—C5	118.1 (2)	O1—C7—H7B	109.5
C1—N1—Cu1	120.86 (18)	H7A—C7—H7B	109.5
C5—N1—Cu1	120.97 (19)	O1—C7—H7C	109.5
N1—C1—C2	122.8 (3)	H7A—C7—H7C	109.5
N1—C1—H1	118.6	H7B—C7—H7C	109.5
C2—C1—H1	118.6		
			
C5—N1—C1—C2	−1.5 (4)	Cu1—N1—C5—C4	−173.5 (2)
Cu1—N1—C1—C2	174.6 (2)	C3—C4—C5—N1	−1.3 (4)
N1—C1—C2—C3	−1.0 (4)	C7—O1—C6—O2	1.3 (5)
C1—C2—C3—C4	2.3 (4)	C7—O1—C6—C3	−178.5 (3)
C1—C2—C3—C6	−177.2 (3)	C2—C3—C6—O2	−3.7 (5)
C2—C3—C4—C5	−1.2 (4)	C4—C3—C6—O2	176.8 (3)
C6—C3—C4—C5	178.3 (3)	C2—C3—C6—O1	176.1 (3)
C1—N1—C5—C4	2.7 (4)	C4—C3—C6—O1	−3.4 (4)

Symmetry codes: (i) −*x*+1, −*y*, −*z*+1; (ii) −*x*+2, −*y*, −*z*+1; (iii) *x*−1, *y*, *z*.

### Hydrogen-bond geometry (Å, º)

**Table:**

*D*—H···*A*	*D*—H	H···*A*	*D*···*A*	*D*—H···*A*
C2—H2···Cl1^iv^	0.95	2.83	3.517 (3)	130
C7—H7*B*···O2^v^	0.98	2.53	3.470 (4)	161
C7—H7*C*···O1^vi^	0.98	2.58	3.298 (4)	130

Symmetry codes: (iv) *x*, *y*, *z*−1; (v) *x*−1/2, −*y*+1/2, *z*+1/2; (vi) *x*−1/2, −*y*+1/2, *z*−1/2.
