# Supplementary figures and images for: Crystal structure of di­chlorido­bis­(methyl isonicotinate-κN)copper(II)
Source: Acta Crystallogr E Crystallogr Commun. 2015 Apr 18;71(Pt 5):m112–3. doi: 10.1107/S205698901500729X (PMC4420097; doi:10.1107/S205698901500729X)

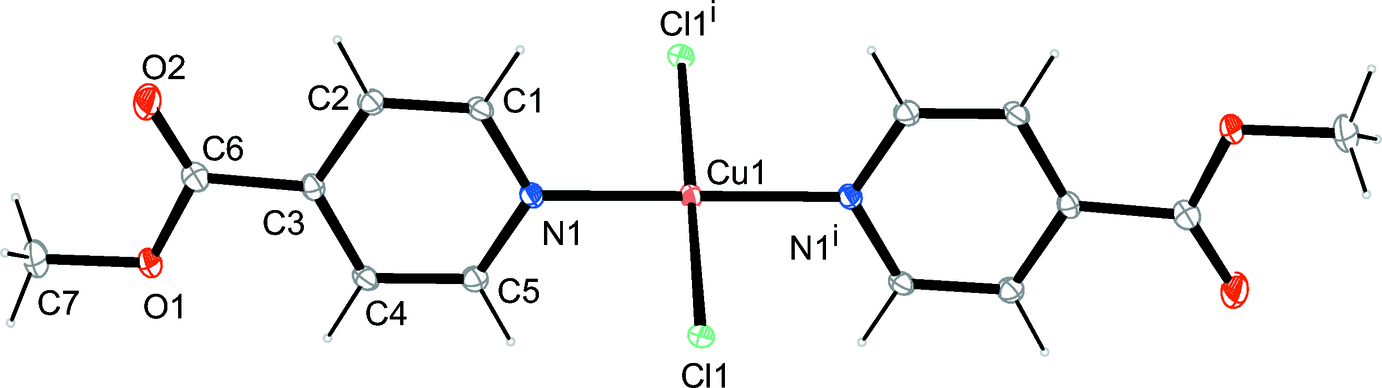

Supplement: Supplementary file 3 [file e-71-0m112-fig1.tif]

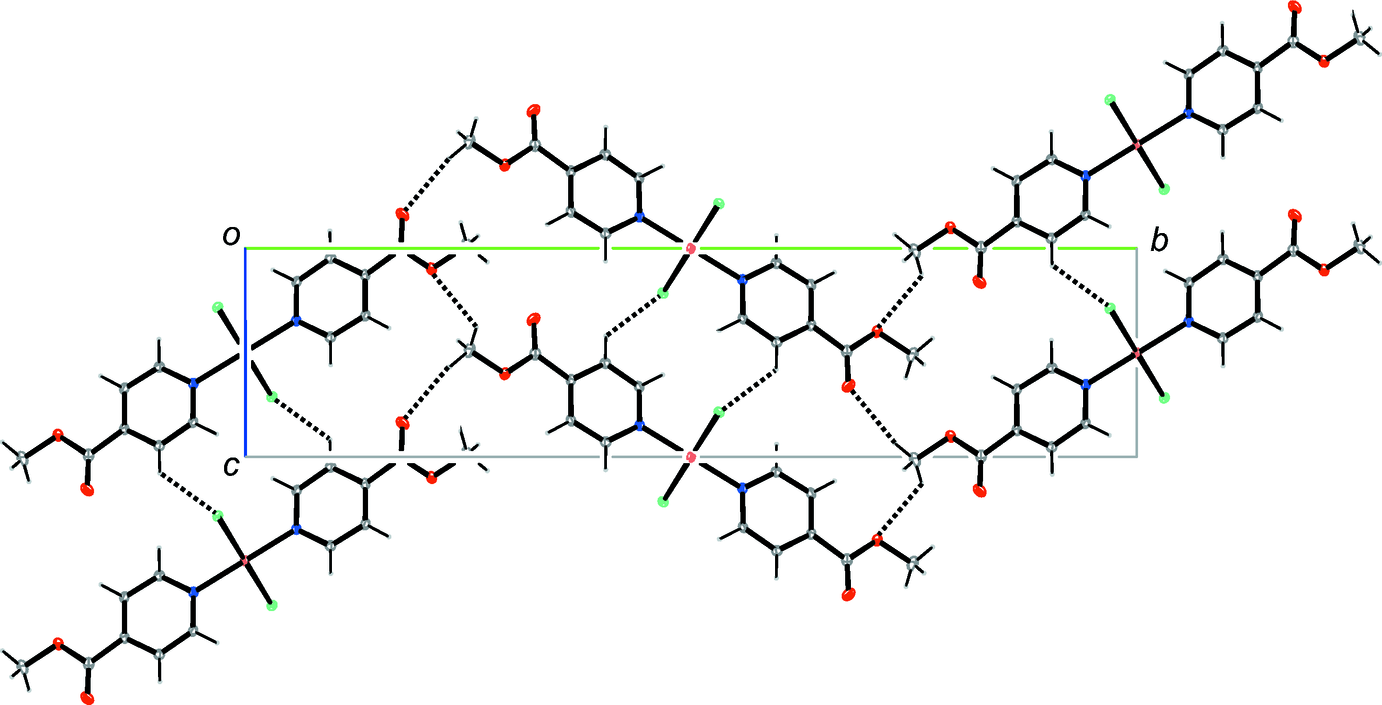

Supplement: Supplementary file 4 [file e-71-0m112-fig2.tif]

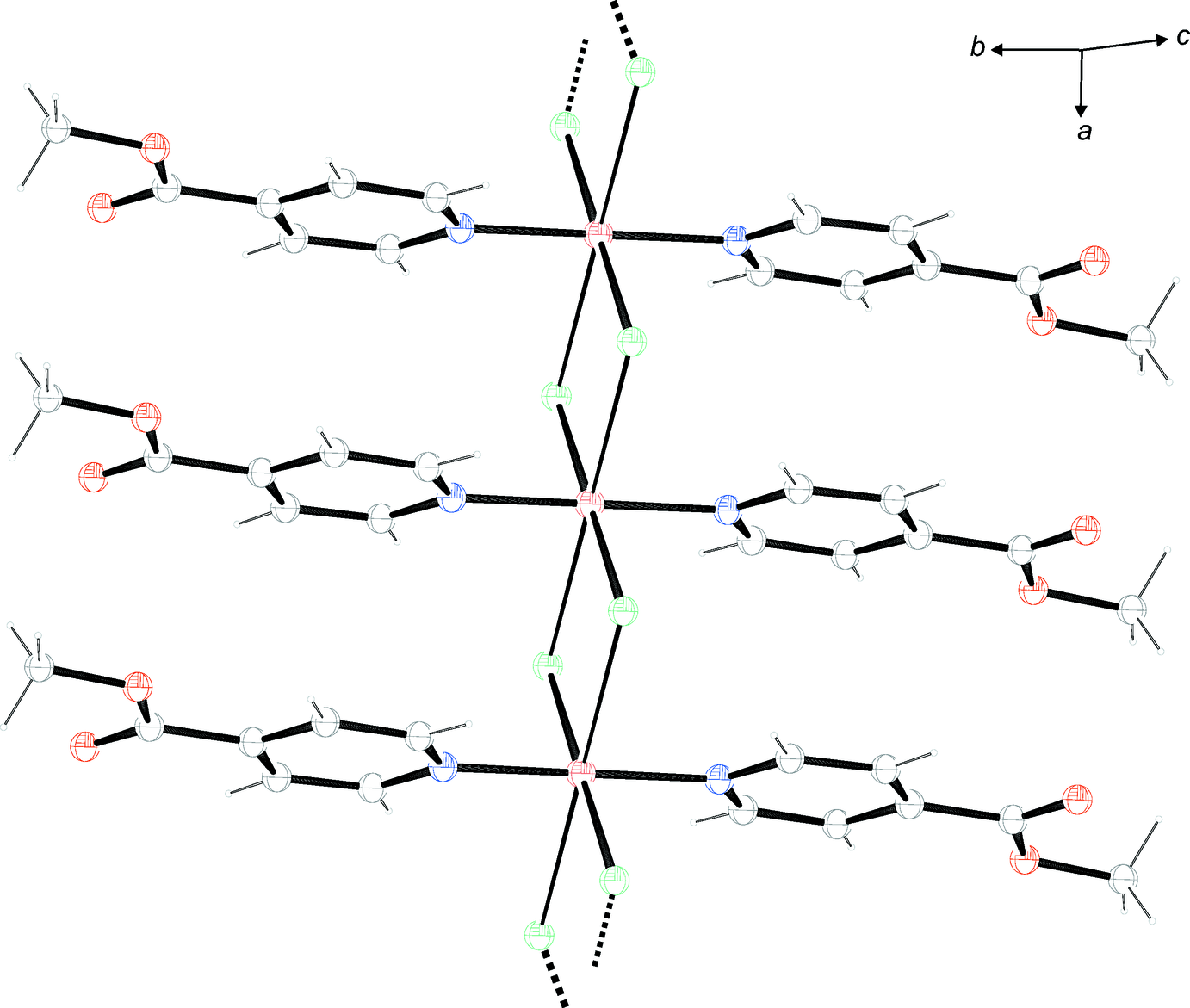

Supplement: Supplementary file 5 [file e-71-0m112-fig3.tif]
